# Revisiting the Origins of Cutaneous T-Cell Lymphoma: A Progenitor-Based Model

**DOI:** 10.3390/cancers18091393

**Published:** 2026-04-28

**Authors:** Yumeng Zhang, Lubomir Sokol

**Affiliations:** Department of Malignant Hematology, H. Lee Moffitt Cancer Center, Tampa, FL 33612, USA; lubomir.sokol@moffitt.org

**Keywords:** CTCL, mycosis fungoides, Sézary syndrome, progenitor model, clonal hematopoiesis, single-cell sequencing, tumor microenvironment

## Abstract

Cutaneous T-cell lymphoma (CTCL), primarily mycosis fungoides (MF) and Sézary syndrome (SS), has long been characterized as a neoplasm of mature memory T cells, based on monoclonal T-cell receptor (TCR) rearrangements and tissue-resident memory (TRM)/central memory (TCM) T-cell phenotypes. This review synthesizes converging population-genetic, multi-omic, and single-cell evidence to argue that this characterization is incomplete and that a progenitor-based model better accounts for the full spectrum of disease biology. We present evidence that initiating mutations arise in hematopoietic stem or early lymphoid progenitor survive thymic selection, and diversify after TCR assembly, resulting in branched evolution across both blood and skin. In SS, paired analyses reveal > 200 shared variants between CD34^+^ progenitors and Sézary cells, as well as signal-joint T-cell receptor excision circle (sjTREC) positivity, providing direct progenitor-level evidence. In MF, convergent signals, multiple malignant clonotypes per lesion, greater blood–skin than skin–skin clonotype overlap, and compartment-specific CNV subclones, implicate hematogenous seeding and reseeding. Population-scale lymphoid clonal hematopoiesis and lymphoid-pattern mosaic chromosomal alterations define a compatible antecedent state. Spatial single-cell atlases and trajectory analyses map site-conditioned programs in skin, including Th2-skewed cytokines, microbial responses, and UV signatures, that select and expand subclones and explain inter- and intra-patient heterogeneity. This framework reconciles mature immunophenotypes with upstream initiation and clarifies why compartment-focused therapies often reshape rather than eradicate disease. It yields testable predictions and actionable implications: trials should pair multicompartment cytoreduction with strategies that attenuate progenitor-derived reservoirs, restore immune balance, and repair skin barrier dysfunction. A progenitor-initiated, niche-adapted model provides a coherent scaffold for more durable control in CTCL.

## Simple Summary

Cutaneous T-cell lymphomas (CTCL), including mycosis fungoides and Sézary syndrome, are complex diseases long thought to arise from mature memory T cells. Recent genomic and single-cell studies suggest a new model in which early mutations occur in hematopoietic stem or lymphoid progenitor cells, long before malignant T cells appear in the skin or blood. This review summarizes key evidence for a progenitor-based origin and explains how it may clarify the disease’s heterogeneity, treatment resistance, and relapse patterns. While direct evidence is currently limited to MF and SS, the progenitor-based framework likely applies to other mature T-cell CTCLs as well, though this remains to be formally tested. Understanding CTCL as a progenitor-initiated disorder may help guide future therapies that target not only skin lesions but also deeper cellular reservoirs to achieve more durable disease control.

## 1. Introduction

Cutaneous T-cell lymphoma (CTCL) is a rare but increasingly common group of T-cell lymphoproliferative disorders that primarily involves skin, with secondary involvement of blood, lymph nodes, and, less frequently, visceral organs in the advanced stage [1,2]. It affects approximately 30,000 patients in North America and showed a 2–3-fold increase in incidence over the past three decades [3,4]. Mycosis fungoides (MF) and Sézary Syndrome (SS) account for about 70% of CTCL [5]. CTCL is considered incurable. Both MF and SS have a high risk of relapse and often require multiple lines of therapy, with a response rate of ~30% and response lasting 6–12 months [2,6,7].

Historically, the cell of origin for CTCL was thought to be mature memory T cells. Early studies demonstrated monoclonal T cell receptor (TCR) rearrangements in the tumor cells using multiplex PCR and bulk sequencing, consistent with transformation of a fully rearranged, mature T cell [8,9]. Immunophenotypic studies reinforced this view. It was hypothesized that MF arises from mutated skin-resident effector memory T (TRM) cells expressing cutaneous lymphocyte-associated antigen (CLA) and CCR4, but lacking L-selectin and CCR7 expression, which would enable them to enter the lymph nodes and blood. SS, a leukemic form of CTCL, was postulated to arise from skin-resident central memory T cells (TCM) that express L-selectin and CCR7 as well as CLA and CCR4, enabling them to infiltrate the skin, lymph nodes, and blood, resulting in greater morbidity and mortality [10,11]. Both MF and SS display a mature T-cell immunophenotype due to the putative malignant transformation of a mature T cell residing in the skin. This concept has been widely accepted because it historically follows our understanding and classification of lymphoid malignancies stratified into immature and mature subtypes.

More recent evidence reframes this view (Table 1). High-throughput and single-cell sequencing demonstrate multiple malignant clonotypes within lesions and greater clonotype overlap between blood and skin than between separate skin sites [12,13,14,15]. Single-cell and spatial multi-omics reveal profound intra- and inter-patient heterogeneity in phenotype and mutational architecture [16,17,18,19]. These findings suggest that monoclonality and mature immunophenotypes describe the final malignant phenotype, but not necessarily the initiating event. The most recent advances in high-throughput and single-cell sequencing suggest that hematologic malignancies might evolve on the basis of clonal hematopoiesis with initiating mutations in bone marrow hematopoietic stem cells. Importantly, these initiating mutations do not cause differentiation arrest but allow mutated cells to complete maturation and acquire the malignant phenotype.

The progenitor-based model reconciles these perspectives by proposing that initiating mutations arise in hematopoietic stem or early lymphoid progenitors, which survive thymic selection and differentiate into diverse mature T-cell subsets. Malignant expansion then occurs in the skin and blood niches, shaped by UV mutagenesis, microbial antigens, and inflammatory cytokines, and later acquires additional changes in the skin and blood microenvironments [16,18,19,20]. This article reviews the relevant evidence supporting this framework and explores its therapeutic and clinical implications.

**Table 1:** Evidence supporting mature T-cell versus progenitor cell origin in CTCL.

Category	Evidence Supporting Mature T-Cell Origin	Evidence Supporting Progenitor Cell Origin
**TCR clonality**	Early studies showed identical TCRγ, β, and α CDR3 sequences in malignant cells, consistent with the transformation of fully rearranged mature T cells [8,9].	High-throughput sequencing revealed multiple malignant clonotypes within lesions and greater overlap between blood and skin than between skin sites, suggesting transformation before full TCR assembly and ongoing reseeding [12,13,14,15].
**Immunophenotype**	MF cells are described as skin-resident effector memory T cells (TRM); SS cells as central memory T cells (TCM) with blood–skin recirculation [10,11].	Immunophenotypic heterogeneity: within the same patient, malignant cells range from naïve to effector memory, central memory, and terminal effector phenotypes, highlighting T cell plasticity [21,22].
**Mutational landscape**	Monoclonality is compatible with both mature transformation and post-thymic differentiation from progenitor-initiated clones.	Shared mutations between hematopoietic progenitors (CD34^+^ cells) and malignant T cells in SS, >200 shared variants [23].
**Clonal evolution**	Reduction in the dominant clone with HDACi treatment further supported a single founding mature T-cell origin [24].	Single-cell and trajectory studies show branching evolution and compartment-specific adaptation of clones [16,17,19]. Xenograft studies show subclonal divergence despite identical TCR rearrangements [25].
**Microenvironment interactions**	Th2 cytokine skewing (IL-4, IL-13, IL-31) was attributed to characteristic mature effector T-cell responses to cutaneous antigenic stimulation [16].	Progenitor-derived clones adapt differently in blood vs. skin; skin niche enriched for UV mutagenesis, microbial antigens, and cytokine signaling that shape malignant expansion [16,26].

## 2. Lymphoid Clonal Hematopoiesis (L-CH) as an Origin of MF/SS

Clonal hematopoiesis (CH) denotes the expansion of hematopoietic stem cell clones bearing somatic mutations in otherwise healthy individuals. When detected incidentally in individuals without cytopenias or a diagnosed hematologic malignancy, this is termed clonal hematopoiesis of indeterminate potential (CHIP), a subclinical state associated with increased risk of hematologic malignancies and cardiovascular disease. While CH was first linked to myeloid neoplasms (e.g., DNMT3A, TET2, IDH1/2, and TP53), population-scale data demonstrate lymphoid clonal hematopoiesis (L-CH) in which driver mutations for lymphoid cancers appear years before diagnosis [27,28]. Critically, L-CH clones can originate in hematopoietic stem cells (HSCs) or committed early lymphoid progenitors. Niroula et al. examined 235 genes that are recurrently mutated in lymphoid malignancies in 46,706 individuals using the UK Biobank and identified 597 individuals (1.3%) carrying different variants of lymphoid driver mutations, which they termed L-CHIP [27]. At a median follow-up of 10 years, they found that L-CHIP was associated with a significantly higher incidence of lymphoid malignancies (HR = 4.2, 95% CI 2.7–6.7), especially chronic lymphocytic leukemia/small lymphocytic lymphoma (CLL/SLL) [27]. Independently, Quijada-Álamo et al. showed identical mutations in CLL cells and CD34+ progenitor cells using next-generation sequencing, suggesting that the initiating mutations in CLL might occur in hematopoietic stem cells [29].

Beyond gene-level L-CH, UK Biobank analyses show that lymphoid-pattern mosaic chromosomal alterations (mCAs), for example, del(11q), del(13q), del(17p), and trisomy 12, predate diagnosis and markedly increase future risk of lymphoid malignancies (especially CLL/NHL) [27,30,31]. These large-scale epidemiological data establish that premalignant lymphoid clones can persist in the marrow compartment for years before clinical disease manifests.

Analogous biology is documented in T-cell lymphomas. In angioimmunoblastic T-cell lymphoma (AITL), a subtype of peripheral T-cell lymphoma, non-tumor marrow and progenitor compartments carry TET2/DNMT3A mutations that are also present in the tumor [32]. Sakata-Yanagimoto et al. identified TET2 and DNMT3A mutations in the non-tumor HSC as well as tumor samples in patients with AITL [32]. Scourzic et al. further demonstrated that sequential transplantation of inactivated TET2-mutated and DNMT3AR882H-mutated progenitor cells led to an AITL phenotype in murine models [33]. Furthermore, Cheng et al. sequenced the HSC and tumor samples from 25 patients with AITL and 2 patients with PTCL-NOS and found that 70.4% of patients had CH-associated genomic alterations, most commonly TET2 and DNMT3A [34]. Collectively, this evidence implies that the initiating mutations of AITL occur in bone marrow as CH, providing an excellent model that T-cell lymphoma can originate in bone marrow but still acquire a mature T-cell lymphoma phenotype.

These population-scale signals do not prove CTCL initiation in marrow but support the concept of a premalignant lymphoid antecedent compatible with a progenitor-initiated trajectory. In MF-spectrum disease, identical malignant TCR clonotypes were detected in bone marrow years before cutaneous disease [35]. Concordant donor–recipient CTCL after allogeneic transplantation further supports transmission of a premalignant/malignant clone via marrow [36]. In addition, the risk of MF/SS is increased in patients with other hematologic malignancies such as CLL or other types of non-Hodgkin’s lymphoma [37,38]. In Sézary syndrome, paired multi-omic analyses reveal >200 shared mutations between CD34^+^ hematopoietic progenitors and Sézary cells, and malignant cells retain signal-joint T cell receptor excision circles (sjTREC), a marker of recent thymic TCR rearrangements, suggesting that Sézary cells are of recent thymic egress and providing direct evidence for progenitor-level initiation [23]. For MF, the evidence remains inferential pending paired marrow–lesion sequencing.

## 3. Thymus Dysfunction in Aging and Its Relevance to CTCL

MF and SS predominantly occur in older individuals, with a median age at diagnosis exceeding 60 years. Age-related thymic involution is therefore a potentially important contributor to disease pathogenesis within the progenitor-based framework. With aging, the thymus undergoes progressive atrophy and functional decline. The thymic output of naïve T cells decreases dramatically, and the diversity of the T cell antigen receptor (TCR) repertoire contracts accordingly, resulting in disrupted T cell homeostasis [39]. Critically, the atrophied thymus continues to produce T cells but with a reduced ability to perform negative selection on self-reactive or mutated T cells, contributing to diminished central tolerance [39].

In the context of the progenitor-based model, thymic aging can potentially contribute to CTCL pathogenesis in several interconnected ways. First, mutated T-cell progenitors derived from bone marrow may have a selective advantage in evading deletion in the dysfunctional thymus. Second, reduced output of functional naïve T cells can promote the oligoclonal expansion of previously generated memory T cells and a contracted TCR repertoire, creating an immunological niche in which mutated clones face less competition. Third, the age-related increase in self-reactive T cells contributes to chronic inflammation and a favorable cytokine microenvironment for tumorigenesis.

Thymic dysfunction intersects with two other age-dependent phenomena: the increasing incidence of lymphoid clonal hematopoiesis of indeterminate potential (L-CHIP) with aging and the chronic low-grade inflammatory state known as inflammaging. Together, thymic involution, age-related inflammation, and accumulating clonal hematopoiesis create a permissive environment that helps explain the characteristically late onset of MF/SS in the patient population. Within the progenitor model, the thymus serves not as the site of malignant transformation but as a checkpoint whose age-related dysfunction permissively favors the survival of the mutated clone. This occurs not because most progenitor-level mutations directly alter TCR-MHC/peptide affinity or signaling thresholds but rather because the atrophied thymus performs negative selection less stringently overall, reducing competitive pressure on all developing clones. Mutated progenitor-derived clones thus escape into the periphery not through selection evasion per se, but through the general erosion of central tolerance and the reduced output of functional naïve T cells that would otherwise compete for survival signals.

## 4. Heterogeneity in MF/SS

### 4.1. Immunophenotypic Heterogeneity

The significant diversity in the immunophenotype of MF/SS is difficult to reconcile with the traditional central dogma. Our group analyzed the immunophenotype of Sézary cells in 52 SS/MF patients [21]. We discovered that the immunophenotype varied significantly within the same patient and that the pattern of the dominant subset of mature T cells did not point to a specific diagnosis, either SS or MF. Both MF and SS patients displayed a broad spectrum of naïve/mature T cell immunophenotypes, including naïve T cells, effector memory T cells, central memory T cells, and terminally differentiated effector memory T cells. The composition of dominant immunophenotypes did not correlate with the clinical diagnosis of MF or SS and did not point to a specific subset of mature T cells as the tumor cell of origin [21]. Moins-Teisserenc et al. independently reported similar results confirming the diversity of SS using the KIR3DL2/CD158k marker, demonstrating unprecedented heterogeneity among circulating malignant cells [22]. Roelens et al. further showed that circulating and skin-derived Sézary cells, while clonal, exhibited phenotypic plasticity [40]. These observations are inconsistent with a single mature T-cell origin but are readily explained by a progenitor-initiated model in which mutated precursors differentiate into multiple mature T-cell subsets.

### 4.2. Clonotypic Heterogeneity

The previous cornerstone evidence supporting MF/SS as a mature T-cell neoplasm came from demonstrations of TCR gene monoclonality using multiplex PCR, high-throughput PCR, and bulk DNA/RNA sequencing [8]. To understand the significance of clonotypic heterogeneity, it is important to review TCR rearrangement biology. TCR δ-, γ-, β-, and α-loci are sequentially rearranged in the thymus using diverse V(D)J gene segment pools, producing a unique CDR3 sequence for each T cell [9]. Each T cell thus carries a unique TCR to interact with antigen, and TCRs (with the notable exception of TCRδ) are retained in all daughter cells. Therefore, identical TCRγ, β, and α sequences in all lymphoma cells would prove that malignant transformation took place in a mature T cell that had completed TCR rearrangement. Conversely, neoplastic cells with different rearrangements in TCR γ-, β-, and α-loci would implicate an immature progenitor prior to thymic rearrangement. Neoplastic T cells sharing the same TCRγ but differing in TCRβ and TCRα sequences would likely derive from a common T-cell precursor at the beginning of or before the thymic stage [41].

Using this framework, Kirsch et al. performed high-throughput sequencing of the TCRγ and TCRβ CDR3 regions in 43 MF/SS patients [12]. They found that the combined abundance of the top two TCRγ clones was similar to the abundance of the top TCRβ clone in the majority of patients, consistent with the expected ratio in mature peripheral blood αβ T cells. However, this relationship holds if MF/SS is a monoclonal disease with a single TCR clone. If MF/SS is a polyclonal disease with one dominant TCR, then additional malignant clones bearing different TCRs would be missed. Notably, the dominant TCR clones accounted for as few as 15% of total T cells, and the remaining 85% were attributed to clonal reactive T cells by the authors [12]. This raised the critical question: could minor malignant T-cell clones with different TCRs be misidentified as part of the reactive T-cell population?

In 2019, Iyer et al. [13] used whole-exome sequencing (WES) and whole-transcriptome sequencing (WTS) of the CDR3 region to directly evaluate this question. They identified multiple TCRγ, TCRα, and TCRβ clonotypes and matched them with atypical lymphocytic infiltrates in laser-captured microdissection areas from 27 patients with MF. Surprisingly, the frequency of the most abundant clones was not correlated with the proportion of tumor cells in the sample, and a single TCRβ clonotype could not account for all malignant cells. Even in samples with perfect TCRγ monoclonality, the dominant TCRβ clone accounted for only approximately 15% of tumor cells. WTS confirmed that malignant T-cell clones harbored multiple TCRγ, TCRα, and TCRβ clonotypes [13]. Iyer et al. further provided evidence of polyclonality, especially in TCRβ and TCRα rearrangements, in a larger cohort [42]. This observation directly questioned the traditional central dogma and supported a model in which initial malignant transformation occurs earlier than or at the level of T-lymphocyte progenitors before TCRα or TCRβ rearrangement, with subsequent hematogenous dissemination to the skin.

Iyer’s group further examined clonotypic heterogeneity by comparing TCR clonotypes between skin lesions and between skin and blood in patients with MF [14]. Extensive clonotypic heterogeneity was observed not only between different lesions but also within individual lesions. There were only a few shared clonotypes, and the shared clonotypes were not always the most frequent. Critically, the degree of clonotypic sharing was higher between skin and blood than between two skin biopsy specimens, leading Iyer et al. to propose a model of consecutive seeding of the skin by neoplastic MF clones circulating in the blood [14]. In addition, transcripts related to activation and cell cycle progression were markedly enriched in malignant cells from the skin compared to the blood, indicating that the skin microenvironment promotes T-cell activation and rapid malignant expansion [17]. Phylogenetic analyses demonstrated branched subclones across compartments [14,15]. Together, these data reconcile the mature immunophenotype with early initiation: initiating mutations precede full TCR assembly, after which malignant clonotypes emerge and seed the skin from blood over time.

### 4.3. Mutational and Transcriptional Heterogeneity

The mutational landscape of CTCL is remarkable for its complexity and inter-patient variability. Wang et al. reported whole-exome sequencing data from 37 patients with SS and identified 4738 somatic mutations, with an average somatic mutation rate of 3.85 mutations/Mb and a nonsynonymous mutation rate of 2.75 mutations/Mb [43]. This mutational burden is similar to that of solid tumors but substantially higher than that of other lymphomas. Strikingly, no common driver mutations were identified. Subsequent large-scale genomic profiling studies identified alterations in several putative oncogenes and tumor suppressor genes, including CARD11, TP53, NFκB pathway components, and JAK/STAT signaling members, but with minimal overlap between studies, highlighting the remarkable inter-patient heterogeneity [44]. Furthermore, variant allele frequencies of mutations differed significantly within the same individual, providing evidence of intra-patient heterogeneity.

Advances in single-cell RNA sequencing (scRNA-seq) have provided a valuable tool for investigating this heterogeneity at higher resolution. Despite small sample sizes, multiple groups demonstrated substantial inter-tumoral and intra-tumoral heterogeneity in surface protein expression and mRNA profiles of MF and SS [24,45,46]. Buus et al. discovered that classical biomarkers of CTCL were heterogeneously expressed within the neoplastic cells of individual patients [24]. Gaydosik et al. identified diverse lymphocyte populations within advanced CTCL skin tumors, underscoring the complexity of the tumor microenvironment [45]. Borcherding et al. separated the malignant population into five transcriptionally distinct clusters and discovered a clonal evolution pattern based on the expression of FOXP3, GATA3, and IKZF2, consistent with clonal diversification [47].

Integrated single-cell and TCR analyses then revealed a pattern of heterogeneity with direct implications for cell of origin: cells sharing identical TCR rearrangements nonetheless harbored distinct CNV profiles across blood and skin compartments, implying divergence from a common progenitor-rooted ancestor after thymic TCR assembly at the latest. Herrera et al. paired scRNA-seq with single-cell TCR analysis and discovered that, even within the same TCR clone, there were distinct CNV-defined subclonal populations in the blood and skin of the same patient [17]. Phylogenetic analysis showed highly branching but shared subclonal evolution in both skin and blood, suggesting continued migration of neoplastic cells between compartments [17]. Iyer et al. independently confirmed the branched relationship pattern between subclones using WES and microdissection techniques [15].

Recent single-cell and spatial studies further strengthen the progenitor-based, site-conditioned model. A spatial single-cell atlas mapped cutaneous niches, including B-cell/dendritic cell aggregates, with malignant programs enriched for Th2/polarized cytokine signaling, microbial-response pathways, and UV-stress signatures [16,18,19,20]. Pseudotime and trajectory analyses demonstrated branching, compartment-dependent evolution and transitional states, with circulating malignant clones diversifying in skin versus blood [17,42]. Complementary xenograft work showed that clonally related Sézary cells diverge under selection, underscoring malignant plasticity [25]. One alternative explanation for the observation of shared somatic mutations across cells with divergent TCR sequences merits brief consideration: TCR editing, whereby mature T cells undergo secondary rearrangement generating a new TCR, could theoretically produce daughter cells with distinct TCRs from a single transformed mature T cell. However, TCR editing is rare in vivo, has not been documented in CTCL, and would require recurrent occurrence across multiple patients in a stereotyped pattern, a highly implausible scenario. The progenitor model, in which mutations arise before thymic TCR assembly and clonotypic diversity emerges during normal T-cell development, provides a far more parsimonious explanation.

Genetic heterogeneity and the absence of common driver mutations do not on their own discriminate between a progenitor and a mature T-cell origin, but they do place important constraints on what any model must explain. In the context of the direct progenitor-level evidence presented above, this mutational architecture is more consistent with a model in which initiating lesions arise early, before TCR assembly, and downstream mutations are acquired independently across subclones in distinct niches.

## 5. New Model for CTCL Pathogenesis and Its Therapeutic Implications

**Figure 1:**
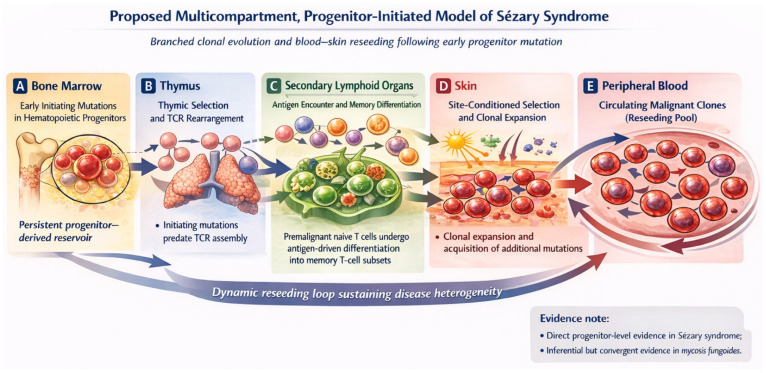
Multicompartment, progenitor-initiated model of Sézary syndrome. Early initiating mutations are proposed to arise in hematopoietic progenitors within the bone marrow, establishing a persistent progenitor-derived reservoir (**A**). These immature cells precede thymic T-cell receptor (TCR) rearrangement and thymic selection, thereby permitting the generation of multiple related descendant T-cell clones (**B**). The premalignant naïve T cells are proposed to traffic to secondary lymphoid organs, where antigen encounter and functional maturation promote differentiation into memory T-cell subsets (**C**). In the skin, local microenvironmental pressures are proposed to drive site-conditioned selection, clonal expansion, and acquisition of additional mutations (**D**). These related malignant clones populate the peripheral blood as a circulating reseeding pool, supporting bidirectional blood–skin trafficking and repeated seeding of cutaneous sites (**E**). The curved arrows denote a dynamic reseeding loop that may sustain spatial and temporal disease heterogeneity. Direct progenitor-level evidence supports this framework in Sézary syndrome, whereas evidence in mycosis fungoides remains convergent but inferential.

Current evidence supports a progenitor-based framework (Figure 1). Like CHIP in myeloid malignancies, initiating mutations of CTCL arise in HSCs or early lymphoid progenitors. However, these mutations do not cause developmental arrest; instead, they allow mutated cells to continue differentiating into T-cell progenitors. Mutated T-cell progenitors migrate to the thymus, where most are deleted during negative selection. The surviving clones undergo TCR gene rearrangement, resulting in diverse mutated T-cell clones in the peripheral blood. In many individuals, immune surveillance restrains these clones, and such patients will not develop clinical manifestations of MF/SS during their lifespan. In others, aging, thymic dysfunction, chronic inflammation, or antigenic stimulation create conditions for further evolution.

SBS7, the hallmark UV mutational signature, reflects C>T transitions from UV-induced DNA damage incompletely repaired by nucleotide excision repair (NER), and dominates in MF relative to SS given its greater cutaneous burden. Within the cutaneous niche, UV exposure leaves the SBS7 mutational signature and *Staphylococcus aureus* superantigens drive polyclonal T-cell activation via JAK/STAT and NF-κB signaling and STAT3 phosphorylation; these do not initiate disease but select and expand progenitor-derived subclones and condition the blood–skin reseeding loop [48,49]. Spatial single-cell maps show Th2-skewed cytokine, microbial-response, and UV-stress programs concentrated in lesional skin niches [16]. A suitable skin microenvironment—antigenic stimulation from skin neoantigens such as the skin microbiome, a favorable cytokine environment, and a pro-inflammatory state related to aging—provides a niche for neoplastic clones to expand. The neoplastic T-cell clones in the blood continue to evolve in a branched pattern by acquiring additional somatic mutations and begin to seed the skin at different sites and time points. After expansion, dominant clones can either stay in the blood or migrate to additional skin sites, lymph nodes, or visceral organs.

Direct evidence supports progenitor-level initiation in SS, while MF currently rests on convergent but inferential data [23,35,36]. For MF, prospective marrow–lesion multi-omics is needed. If initiation occurs in progenitors, we expect low-frequency CTCL-like mutations in CD34^+^ marrow from MF patients and longitudinal detection of evolving UV-SBS7–positive subclones that reseed skin [26,50]. It bears emphasis that UV mutagenesis and *S. aureus* colonization are site-conditioned selective pressures; they shape which progenitor-derived subclones expand in skin, but do not generate the upstream malignant reservoir from which the skin is continuously reseeded.

### Therapeutic Implications of the New Model

This cell-of-origin framework explains both the clinical behavior of MF/SS and their resistance to therapy. Despite significant efforts in treating CTCL, no cure has been discovered. Profound lymphocyte depletion in the skin (e.g., by electron beam radiation therapy), intensive chemotherapy, or targeted therapies provide only a short duration of response. Conventional treatments, whether skin-directed radiation, phototherapy, targeted therapy, or systemic chemotherapy, act on or emphasize one anatomical compartment but fail to eradicate the full skin–blood–marrow system. Similarly, most agents target one specific pathway active in dominant clones but do not address the upstream biology of clonal hematopoiesis or the immune dysregulation that favors repeated reseeding. A critical question emerges from the progenitor model: are current therapies targeting all cell populations with malignant potential, or only the dominant clones?

Single-cell studies illustrate how therapy can reshape rather than eliminate disease. Buus et al. identified that, despite an overall reduction in the number of malignant T cells, some subclones of Sézary cells became resistant after treatment with histone deacetylase inhibitors (HDACi) [24]. Borcherding et al. also discovered the emergence of new transcriptional clusters following HDACi treatment and subsequent hyperexpanded clusters enriched for transcriptional pathways involving amino acid metabolism, DNA repair, and cytokine signaling [51]. The current clinical response criteria are based predominantly on the evaluation of dominant neoplastic clones; minor subclones that can serve as a reservoir for future relapses and resistance are typically missed.

Durable disease control will require approaches that go beyond single compartments or dominant clones. Because UV/SBS7- and *S. aureus*–driven programs mark site-conditioned selection rather than initiation, durable control will require multicompartment cytoreduction together with immune rebalancing and skin barrier repair to reduce antigenic drive and disrupt reseeding. Restoring immune function not only strengthens tumor surveillance but also counters the Th2-skewed cytokine milieu, reversing age-related immune decline and reinvigorating cytotoxic T cell and NK cell responses. Repairing skin barrier function is equally critical, as barrier disruption permits colonization by *Staphylococcus aureus* and other microbes that provide persistent antigenic stimulation and promote malignant expansion [48,49]. Ultimately, CTCL treatment failure reflects the persistence of a marrow-derived reservoir and the failure to disrupt the cycle of reseeding across skin and blood. Future strategies will likely require a multistep approach that combines reducing disease burden with interventions to restore immune balance and skin barrier integrity to achieve sustained remission.

## 6. Unanswered Questions and Future Directions

Although the progenitor-based model provides a coherent framework for understanding CTCL pathogenesis, several fundamental questions remain unresolved, and their answers will determine whether this paradigm can be translated into clinically meaningful advances.

### 6.1. Does Mycosis Fungoides Definitively Originate from Hematopoietic Progenitors?

Direct progenitor-level evidence is now established in SS. In MF, however, the case currently rests on convergent yet inferential signals: multiple malignant clonotypes within individual lesions, greater blood–skin than skin–skin clonotype overlap, and compartment-specific copy-number variant (CNV) subclones [13,14,15,17,42]. These observations are consistent with hematogenous seeding but do not constitute direct proof. Prospective paired marrow–lesion analyses in MF, analogous to those performed in SS, are needed to determine whether initiating mutations are indeed present in CD34^+^ progenitors and whether the phylogenetic architecture mirrors the branched, progenitor-rooted topology observed in Sézary syndrome.

### 6.2. What Is the Timing and Order of Initiating Versus Niche-Acquired Mutations?

Even if a progenitor origin is confirmed in MF, disentangling mutations acquired in progenitors from those selected within the cutaneous niche remains a central challenge. UV-associated mutational signatures such as SBS7, which dominate in MF but are less prominent in SS, likely represent late, niche-conditioned events rather than initiating lesions. Distinguishing early drivers from late selectors will require temporally resolved, multicompartment sequencing at diagnosis and across disease evolution. Such data would clarify the minimal set of progenitor-level alterations sufficient to confer premalignant potential and identify the niche-derived selective pressures that convert premalignant clones into overt disease, thereby enabling targeted interception strategies.

### 6.3. Distinguishing Benign Lymphoid Clonal Hematopoiesis from CTCL-Predisposing Clones

Population-scale studies have demonstrated that lymphoid clonal hematopoiesis (L-CH) and lymphoid-pattern mosaic chromosomal alterations (mCAs) precede lymphoid malignancies by years [27,30,31]. However, these associations have been established primarily for CLL and NHL [27]; the specificity of L-CH for CTCL is unknown. Critical gaps remain: which molecular features, TCR repertoire characteristics, and microenvironmental correlates distinguish indolent L-CH clones from those predisposed to CTCL transformation? Addressing this question will require large-scale prospective cohorts that integrate deep sequencing of progenitor compartments with long-term clinical follow-up, ultimately defining biomarker panels capable of risk-stratifying individuals harboring premalignant lymphoid clones.

### 6.4. Necessary Versus Permissive Microenvironmental Features for Malignant Selection in Skin

Spatial single-cell atlases have mapped the lesional niches of CTCL and identified enrichment for Th2-skewed cytokines, microbial antigens (notably *Staphylococcus aureus*), B cell/dendritic cell aggregates, and UV-stress signatures. What remains unresolved is the causal hierarchy among these elements: which microenvironmental features are strictly necessary for malignant transformation and clonal expansion, which are merely permissive, and how do they interact? For instance, does *S. aureus* colonization precede and drive Th2 polarization, or does a pre-existing Th2 milieu facilitate bacterial colonization that subsequently amplifies clonal expansion? Do B cell/DC aggregates serve as antigen-presenting niches that sustain malignant T-cell proliferation, or are they reactive bystanders? Functional perturbation experiments in organoid and humanized models, combined with longitudinal spatial profiling of pre-lesional and lesional skin, will be essential to resolve these interdependencies.

### 6.5. Determinants of Blood–Skin Reseeding and Therapeutic Escape

The progenitor model predicts a continuous reseeding loop in which malignant clones circulate between marrow, blood, and skin, with clonal composition dynamically reshaped by therapy [14,17]. Yet the molecular determinants governing compartment tropism—trafficking receptors, chemokine gradients, and metabolic states that dictate homing and retention in skin versus blood—remain incompletely mapped. Equally important, the mechanisms by which subclones escape therapy-imposed selection are poorly understood [24,51]. Serial clonal tracking using paired scTCR-seq and CNV inference across treatment transitions would reveal how specific therapies reshape clonal architecture, whether resistant clones pre-exist or emerge de novo, and which compartments harbor therapy-refractory reservoirs. Such data would directly inform rational sequencing and combination of therapies designed to disrupt the reseeding cycle.

## 7. Conclusions

Emerging genomic and single-cell data support a progenitor-based model in which early initiating mutations occur in hematopoietic stem or lymphoid progenitors. These mutated but not yet malignant clones survive thymic selection, circulate as diverse subclones, and, upon further evolution in the skin niche, undergo malignant transformation and repeatedly reseed the skin. Within the cutaneous niche, UV mutagenesis, microbial antigens, and inflammatory cytokines shape clonal evolution, sustain disease heterogeneity, and confer resistance to compartment-limited therapy. This framework explains frequent relapse after compartment-focused therapies and supports multistep strategies that reduce tumor burden and restore immune balance and skin barrier function, thereby disrupting reseeding and achieving durable remission.

Several emerging data streams will be critical to advancing this framework. Prospective paired marrow–lesion multi-omic studies in MF, analogous to those already performed in SS, are needed to establish whether progenitor-level initiation is a universal feature of CTCL. Single-cell and spatial technologies applied longitudinally—at diagnosis, across therapy, and at relapse—will map how the progenitor-derived reservoir evolves under selective pressure and identify which compartments harbor therapy-refractory clones. Population-scale studies integrating deep progenitor sequencing with long-term clinical follow-up will define the molecular features that distinguish indolent L-CH from CTCL-predisposing clones, enabling risk stratification and early interception.

Therapeutically, the progenitor model reframes the goal of treatment from compartment-focused cytoreduction to multicompartment reservoir elimination. Rational combination strategies that pair skin-directed and systemic cytoreduction with agents targeting progenitor-derived reservoirs, restoring immune surveillance, and repairing skin barrier dysfunction represent the most promising path toward durable disease control. As single-cell, spatial, and longitudinal genomic data continue to accumulate, the progenitor-based framework provides a coherent scaffold for translating mechanistic insights into clinical advances in CTCL.

## Data Availability

The original contributions presented in this study are included in the article. Further inquiries can be directed to the corresponding author.

## Abbreviations

**Abbreviation**	**Definition**
AITL	Angioimmunoblastic T-cell lymphoma
ALCL	Anaplastic large cell lymphoma
CH	Clonal hematopoiesis
CHIP	Clonal hematopoiesis of indeterminate potential
CLA	Cutaneous lymphocyte-associated antigen
CLL	Chronic lymphocytic leukemia
CNV	Copy number variant
CTCL	Cutaneous T-cell lymphoma
HDACi	Histone deacetylase inhibitor
HR	Hazard ratio
HSC	Hematopoietic stem cell
L-CH	Lymphoid clonal hematopoiesis
L-CHIP	Lymphoid clonal hematopoiesis of indeterminate potential
mCA	Mosaic chromosomal alteration
MF	Mycosis fungoides
NER	Nucleotide excision repair
NHL	Non-Hodgkin lymphoma
NK	Natural killer
PTCL	Peripheral T-cell lymphoma
SBS7	Single base substitution signature 7
scRNA-seq	Single-cell RNA sequencing
sjTREC	Signal-joint T-cell receptor excision circle
SLL	Small lymphocytic lymphoma
SS	Sézary syndrome
TCM	Central memory T cell
TCR	T-cell receptor
TRM	Tissue-resident memory T cell
UV	Ultraviolet
WES	Whole-exome sequencing
WTS	Whole-transcriptome sequencing
